# Infection Strategies of Intestinal Parasite Pathogens and Host Cell Responses

**DOI:** 10.3389/fmicb.2016.00256

**Published:** 2016-03-03

**Authors:** Bruno M. Di Genova, Renata R. Tonelli

**Affiliations:** ^1^Departamento de Microbiologia e Imunologia, Universidade Federal de São PauloSão Paulo, Brazil; ^2^Instituto de Ciências Ambientais, Químicas e Farmacêuticas, Departamento de Ciências Biológicas, Universidade Federal de São PauloDiadema, Brazil

**Keywords:** protozoan parasites, intestinal infection, diarrhea, gastrointestinal tract, intestinal epithelial barrier, parasite–host interaction

## Abstract

*Giardia lamblia*, *Cryptosporidium* sp., and *Entamoeba histolytica* are important pathogenic intestinal parasites and are amongst the leading causes worldwide of diarrheal illness in humans. Diseases caused by these organisms, giardiasis, cryptosporidiosis, and amoebiasis, respectively, are characterized by self-limited diarrhea but can evolve to long-term complications. The cellular and molecular mechanisms underlying the pathogenesis of diarrhea associated with these three pathogens are being unraveled, with knowledge of both the strategies explored by the parasites to establish infection and the methods evolved by hosts to avoid it. Special attention is being given to molecules participating in parasite–host interaction and in the mechanisms implicated in the diseases’ pathophysiologic processes. This review focuses on cell mechanisms that are modulated during infection, including gene transcription, cytoskeleton rearrangements, signal transduction pathways, and cell death.

## Introduction

Intestinal infection is the most common cause of diarrhea in humans worldwide and although presenting low mortality rates, complications are not uncommon, with some cases requiring hospital care. Diarrhea may be caused by viruses, bacteria, helminths and protozoa, most of which are disseminated with feces-contaminated water and food. Amongst protozoan parasites, *Giardia lamblia*, *Cryptosporidium parvum*, and *Entamoeba histolytica* are the three most common etiological agents of diarrhea and other related diseases (giardiasis, cryptosporidiosis, and amoebiasis, respectively) characterized as acute and self-limited dysentery. Nevertheless, in some patients disease may become chronic with long-term effects such as malnutrition, growth delays, and cognitive impairment.

Diarrhea is an increase in the volume or liquidity of stool and it may or may not be accompanied by frequent evacuations (Viswanathan et al., 2009). Disorders of both the small and large intestines can result in diarrhea which, based on the duration, may be classified as acute (≤14 days), persistent (from 15 to 29 days) or chronic (≥30 days). This classification is clinically important to determine the etiological agent for diagnostic and treatment purposes (Guerrant et al., 2001). Diarrhea may also be classified into five categories based on the pathophysiological mechanisms as osmotic, secretory, exudative, inflammatory and resulting from motility disturbances (Field, 2003). Osmotic diarrhea is triggered when healthy individuals (with normal gut functions) ingest large amounts of poorly absorbed substrates, usually carbohydrates (polyethylene glycol, mannitol, lactulose) and divalent ions (MgSO_4,_ MgOH_2_; Hammer et al., 1989; Izzo et al., 1994). An increase in intraluminal unabsorbed nutrients associated with epithelial damage and reduction of the intestinal absorptive surface also characterizes osmotic diarrhea. Secretory diarrhea results from overstimulation of intestinal tract secretory capacity. Exposure to enterotoxins from several types of bacteria (e.g., *Escherichia coli* heat-labile toxin, *Cholera* toxin), excessive bile acid synthesis, low levels of short-chain fatty acids and intestinal inflammation (seen in autoimmune diseases like inflammatory bowel disease and celiac disease) can trigger this type of diarrhea (Sullivan et al., 2009; Walters et al., 2009; Binder, 2010). When the intestinal barrier is compromised due to loss of epithelial cells or disruption of tight junctions (TJs), diarrhea is referred as exudative. Finally, increased or decreased propulsion of stools relates to diarrhea caused by motility problems (Field, 2003).

**FIGURE 1:**
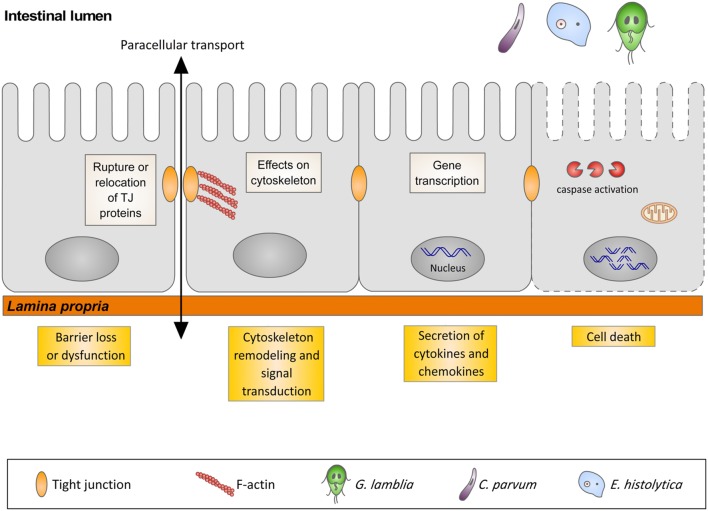
**Illustration representing the interaction of *G. lamblia*, *C. parvum* and *E. histolytica* with the intestinal epithelium and the host cells responses**.

In many gastrointestinal infectious diseases, more than one of the five pathophysiological mechanisms is involved in the development of diarrhea. This is the case for giardiasis, cryptosporidiosis, and amoebiasis that, in spite of sharing similar pathophysiological mechanisms of diarrhea, have different initiating events. The early events triggered by the interaction of these three protozoans with their respective hosts are the focus of this review, with special attention to gene transcription, signal transduction pathways, cytoskeleton rearrangements, and cell death in host cells (**Figure 1**).

## *Giardia lamblia* And Giardiasis

The genus *Giardia* comprises many species that inhabit the intestinal tract of a series of vertebrate hosts including domestic animals, rodents, dogs, cats, livestock, and wildlife. However, one species, *G. lamblia* (synonyms *G. duodenalis* and *G. intestinalis*), is known to infect and cause giardiasis in humans and mammals, suggesting a zoonotic transmission (Ryan and Cacciò, 2013).

*Giardia lamblia* has a simple lifecycle comprising two morphogenetic stages, the infectious and environmentally resistant cyst stage and the vegetative trophozoite stage, which colonizes the small intestine epithelium and causes the disease. Infection initiates when a host ingests viable cysts directly or with contaminated water and food (the infective dose for a symptomatic infection is about 10–100 cysts). After passing through the stomach, cysts begin excysting (excystation process), releasing two trophozoites in the upper part of the small intestine where they adhere to the cells lining the intestinal lumen (enterocytes) through an adhesive ventral disk and multiply by binary fission. Under suitable environmental conditions (i.e., increased bile salt concentration and cholesterol deprivation), trophozoites transform into cysts (encystation process) that are excreted and passed with the feces, thus completing their lifecycle (Gillin et al., 1988; Luján et al., 1996).

### Host Cell Transcription during *Giardia lamblia* Infection

As mentioned, *G. lamblia* trophozoites are confined to the lumen of the intestinal tract of humans and animals. To cause disease they must “swim” through the intraluminal fluid flow, overcome peristaltic motions, evade host immunological defense mechanisms and replicate attached to the small intestine mucosal surface. Colonization of the host intestinal epithelium by trophozoites is mediated by an adhesive, microtubule-based organelle denominated the ventral disk (Holberton, 1973; Adam, 2001; Schwartz et al., 2012). Specific molecular mechanisms may be involved, with a range of *Giardia* cell-surface constituents, including lectins and saccharides, being described as ligands for host cell attachment (Farthing et al., 1986; Inge et al., 1988; Ward et al., 1988; Céu Sousa et al., 2001). Membrane rafts present at the surface of trophozoites are also implicated in trophozoite adhesion to human enterocyte-like cells, as parasite treatment with methyl-β-cyclodextrin (a lipid raft disorganizing agent) resulted in diminished attachment of *G. lamblia* to Caco-2/TC7 cells (Humen et al., 2011). Independent of whether adhesion results from a mere mechanical adhesion through the ventral disk or involves ligand–receptor interactions, the host cells are not passive recipients for *Giardia* attachment but are active participants, having evolved specialized strategies to resist infection. These include, for example, the amplification and regulation of the expression of genes, many of which are involved in the immunological defense of host cells to *Giardia* (Ferella et al., 2014). The importance of the early transcription of genes coding for components of intestinal mucosal immunity can be appreciated by studies using animal models. For example, it has been shown that infection of C57BL/6 mice with *G. muris* induces upregulation of interleukin 17A starting 1 week post-infection (Dreesen et al., 2014). The immune-modulating cytokine interleukin-6 (IL-6) is also involved in the control of *G. lamblia* infection, as IL-6-deficient mice were not able to handle the acute phase of the disease and developed chronic giardiasis (Zhou et al., 2003). Importantly, reverse transcription-PCR-based quantitation of cytokine mRNA levels in peripheral lymph node cells exhibited a short-term upregulation of IL-4 expression in IL-6-deficient mice that seemed to be associated with failure to control the parasite population (Zhou et al., 2003).

Recently, host gene expression of mice whole small intestinal tissue following *G. lamblia* infection has been analyzed using oligonucleotide arrays (Tako et al., 2013). The results from this analysis indicated that genes associated with antibodies, mast cell proteases and matrix metalloprotease 7 (Mmp7) were upregulated (Tako et al., 2013). The role of Mmp7 was then confirmed *in vivo*, as Mmp7-deficient mice presented increased numbers of trophozoites in the small intestine when compared to control animals (Tako et al., 2013). In mice, the *Mmp7* gene encodes the processing proteinase of murine Paneth cell defensins, a class of antimicrobial peptides important in the innate immune response of the small intestines (Ostaff et al., 2013). *G. lamblia* trophozoites are lyzed by α-defensin peptides *in vitro* (Aley et al., 1994), making it plausible to consider that Mmp7-deficient mice were unable to clear the infection with *Giardia* probably due to their inability to release or to process antimicrobial defensins from intestinal epithelia.

*In vitro* as well as *in vivo* models for genome-wide analysis of gene expression have also contributed to understanding the epithelial cell response to *G. lamblia* infection. Using differentiated Caco-2 human cell line as a model of the intestinal epithelium, Roxström-Lindquist et al. (2005) demonstrated that co-incubation of cells with *G. lamblia* resulted in the upregulation of genes coding for chemokines (CCL2, CCL20, CXCL1, CXCL2, CXCL3) and stress-induced genes like *c-Fos*, *c-Jun* and immediate-early response 3 (*IER3*), to cite a few examples. In addition, genes involved in cellular proliferation (*G0S2*, *PCNA*, *ORC5L*, *MCM2*, *MCM3*) had their expression reduced after 6 and 18 h of co-incubation of host cells with trophozoites. Therefore, it appears that infection with *Giardia* interferes with the transcription of host genes involved in innate immunity and prevents cell turnover, probably to maintain a stable niche for colonization as suggested by Stadelmann et al. (2012).

### *Giardia lamblia* Infection and Host Cell Death

At a certain point, the physical attachment of trophozoites to epithelial cells may target specific signaling networks, provoking downstream events that impair normal organ function and lead to associated signs and symptoms of giardiasis. In this scenario, the most striking outcome of *Giardia*–host interaction may be considered the activation of cell death mechanisms such as apoptosis. Apoptosis is described as a regulated and controlled process of autonomous cell death that avoids eliciting inflammation (Elmore, 2007). It can be activated extrinsically by receptor-mediated signaling (through death receptor ligation and DISK assembly) or intrinsically by disruption of the mitochondria. In the first case, the major effectors of apoptosis are a family of aspartic acid-specific proteases known as caspases (Thornberry and Lazebnik, 1998). These are normally synthesized as inactive precursors, but become activated at the onset of apoptosis by activation signals. At the initiation of apoptosis, different sets of caspases are activated depending on whether cell death was triggered extrinsically (caspases 8 and 10) or intrinsically (caspases 9 and 2). However, propagation of the apoptosis signal relies on the direct cleavage of a downstream effector caspase such as caspase-3 that, in turn, cleaves key substrates, producing many of the cellular and biochemical events of apoptosis (Thornberry and Lazebnik, 1998; Slee et al., 2001; Elmore, 2007).

In the gastrointestinal tract, epithelial cells are organized as a single cell layer that covers the entire tissue. They are originated at the crypt and migrate up to the villous tip where they are constantly renewed by extrusion to the lumen. At this point, the turnover of cells is extremely fast (5–7 days) and crucial for the maintenance of normal organ morphology and function. A number of studies have demonstrated that this process is highly regulated and can only be maintained by balancing the levels of cell death and proliferation (Günther et al., 2013). In the gut, cell death occurs through the activation of apoptosis and, although important for gut homeostasis, excessive enterocyte death has been associated with different disorders of the gastrointestinal tract including ulcerative colitis, celiac disease and Crohn’s disease (Ciccocioppo et al., 2001; Di Sabatino et al., 2003; Turner, 2009). Infection of human ileocecal adenocarcinoma cell line HCT-8 with *G. lamblia* can also induce host cell apoptosis. In this case, signs of chromatin condensation were observed within the nuclei of intestinal cell monolayers exposed to different *G. lamblia* assemblages (A, B, and E) or with a combination of these assemblages (Koh et al., 2013). In addition, caspase-3 activation was found to occur in *G. lamblia*-induced apoptosis, as nuclear fragmentation and cell death was effectively suppressed by a caspase-3 inhibitor (Koh et al., 2013). Interestingly, the apoptotic response elicited by *Giardia* was not dependent on co-incubation of host cells with live trophozoites, as sonicated parasites induced caspase-3-dependent apoptosis of non-transformed human duodenal epithelial cell line (SCBN; Chin et al., 2002). Confirming these data, morphological changes consistent with apoptosis and activation of caspase-3 were also observed by others (Panaro et al., 2007). The results from this work indicated that initiation of apoptosis occurred through the activation of caspase-8 and caspase-9, demonstrating that both the intrinsic and extrinsic pathways were triggered during *Giardia* infection (Panaro et al., 2007). At the same time, a significant downregulation of Bcl-2 and increased expression of the pro-apoptotic protein Bax were observed, being the first demonstration of the participation of members from the Bcl-2 family in the induction of enterocyte apoptosis during *Giardia*–host cell interaction (Panaro et al., 2007). Bcl-2 family proteins, which have either pro-apoptotic (Bax and Bak) or anti-apoptotic activities (Bcl-2, Bcl-X_L_, and induced myeloid leukemia cell differentiation protein 1 or Mcl-1), are critical regulators of caspase activation and apoptosis (Adams and Cory, 1998, 2001). In mammalian cells, overexpression of Bax was associated with loss of mitochondrial membrane potential, cytosolic accumulation of cytochrome c, caspase activation, cleavage of poly(ADP-ribose)-polymerase (PARP), DNA fragmentation, and cell death (Oliver et al., 1998). Interestingly, during host cell infections with *G. lamblia*, cleavage of PARP occurred upon activation of caspase-3 (Panaro et al., 2007). Taken together, these data indicate that downregulation of Bcl-2 and upregulation of Bax is associated with the activation of both the intrinsic and extrinsic apoptotic pathways during *G. lamblia* infection. In this scenario, the activated initiators caspase-8 and caspase-9 trigger executioner caspase-3, leading to the proteolytic cleavage of PARP and induction of apoptosis (Panaro et al., 2007).

While the critical role of apoptosis in the pathophysiology of giardiasis is well-documented, host responses to prevent cell death during *Giardia* infection remain poorly understood. In an attempt to shed light on this issue, some authors postulated that inhibition of apoptosis in *Giardia*-infected cells would involve the upregulation of sodium-dependent glucose cotransporter (SGLT)-1 as demonstrated by bacteria. In this work, Yu et al. (2005) described that activation and enhanced glucose uptake into enterocytes rescued cells from lipopolysaccharide (LPS)-induced apoptosis via SGLT-1. In *G. lamblia* infections, it was shown that, when SGLT-1-transfected Caco-2 cells were exposed to trophozoite products in high (25 mM) glucose media, host cell apoptosis was abolished (Yu et al., 2008). In addition, a soluble proteolytic fraction of *G. lamblia* was found to upregulate SGLT-1-mediated glucose uptake in association with increased apical SGLT-1 expression in epithelial cells (Yu et al., 2008). These findings indicated that SGLT-1-dependent glucose uptake might represent a novel epithelial cell rescue mechanism against *G. lamblia*-induced apoptosis.

### Disassembly of Tight Junctions and Cytoskeleton Reorganization during *Giardia lamblia* Infection

The intestinal epithelium is formed by a single layer of epithelial cells that function as a physical barrier between the lumen and the subepithelial tissue. It prevents the entrance of microorganisms, luminal antigens, and toxins to the mucosal tissue; controls the paracellular movement (transport in the space between epithelial cells) of water, solutes, and macromolecules; and regulates cell proliferation, polarization, and differentiation (Turner, 2009). Within the barrier, epithelial cells are held together by complex structures of tetraspan (claudins, occludins, and tricellulin) and single-span transmembrane proteins known as TJs or zonulae occludentes (ZOs; Balda and Matter, 2008; Turner, 2009). TJs form a continuous belt-like structure that completely encircles the cell at the apical region of the plasma membrane. They also work as signaling platforms, as junctional proteins like ZO-1 interact with cytoskeleton actin through a plaque of cytoplasmic proteins localized under the junction (Matter and Balda, 2003; Turner, 2009). Due to their importance in providing adhesive contacts between neighboring cells and in controlling the permeability of the intestinal barrier, it is not surprising that disruption or reduced expression of TJ proteins and loss of epithelial barrier function have been associated with many intestinal disorders including giardiasis (Teoh et al., 2000; Buret et al., 2002; Chin et al., 2002; Müller and von Allmen, 2005; Buret, 2007; Troeger et al., 2007; Koh et al., 2013). Indeed, some *in vitro* studies demonstrated that co-incubation of different cell lines (SCBN and HCT-8) with trophozoites results in the disruption of the TJ protein ZO-1 which, in some cases, leads to increased cell permeability (Buret et al., 2002; Chin et al., 2002; Koh et al., 2013). Disruption of ZO-1 by *Giardia* was associated with caspase-3-dependent apoptosis, as the loss of this protein was abolished in cells treated with caspase-3 inhibitors prior to infection (Chin et al., 2002; Koh et al., 2013). In line with these observations, apoptosis has been observed in many diseases involving TJ disruption (Zeissig et al., 2007; Su et al., 2013). However, whereas in some of these diseases apoptosis is a downstream response to loss of junctional proteins, in giardiasis this fact has yet to be determined.

In recent years, a body of evidence has indicated that loss of TJs may result not only from reduced expression of junctional components but also as a consequence of their relocation/reorganization within cells. For example, in Alzheimer’s disease, it was shown that endothelial cells exposed to β-amyloid peptide (Aβ) display a disrupted plasma membrane pattern of claudin-5 and ZO-2, which are relocated to the cytoplasm (Marco and Skaper, 2006). During enteropathogenic *E. coli* infections, loss of occludin association with claudin-1 and ZO-1 was observed to occur due to the translocation of apically localized TJ proteins to the lateral membrane (Muza-Moons et al., 2004). In this case, the *E. coli*-induced reorganization of junctional complexes resulted in decreased transepithelial electrical resistance (TEER) and disruption of the intestinal barrier (Muza-Moons et al., 2004). In *G. lamblia* infections, relocation of TJ proteins ZO-1 and claudin-1 from the cell–cell contact region to the cytoplasm were shown to occur during co-incubation of Caco-2 cells with trophozoites. Moreover, F-actin was retracted and concentrated near cellular contacts, resulting in microvillous atrophy as observed by scanning electron microscopy (Maia-Brigagão et al., 2012). This is in accordance with previous observations describing that infections of colonic cells with *Giardia* induced localized condensation of F-actin, loss of perijunctional α-actinin and increased cell permeability (Teoh et al., 2000). The mechanism by which epithelial TJs and cytoskeleton were disassembled involved the post-translational modification of the myosin light chain (MLC) of myosin II by MLC kinase (MLCK), as exposition of cells with *Giardia* triggered MLC phosphorylation (Scott et al., 2002). The importance of MLCK in TJ and cytoskeleton disassembly was further reinforced by the observation that co-incubation of cells with a specific MLCK inhibitor blocked the effects of *Giardia* on epithelial permeability, F-actin, and ZO-1 (Scott et al., 2002).

The hypothesis that TJ disruption, either by reduced expression or relocalization of junctional components, has an important role in barrier function in giardiasis is supported by *in vivo* data. In this case, analysis of duodenal biopsy specimens from patients with chronic giardiasis has shown reduced claudin-1 expression and serious villous shortening (Troeger et al., 2007). These were accompanied by a decrease in the absorptive capacity of the duodenum and active anion secretion as evidenced by reduced Na^2+^-coupled D-glucose absorption and electrophysiological measurements, respectively (Troeger et al., 2007).

Therefore, it can be concluded that the activation of the MLCK signaling pathway during giardiasis relates to loss of TJ proteins, cytoskeleton rearrangement and barrier dysfunction, which can contribute to the pathophysiological mechanisms underlying diarrhea such as electrolyte secretion and malabsorption. A summary of the studies about the pathophysiology of *Giardia* infections is listed in **Table 1**.

**Table 1:** Summary on studies describing the effect of *Giardia lamblia* infection on host cell responses.

Target on host cells	Effect	Reference
***Gene transcription***		
Induction of IL-17	Protective immune response against *G. muris* infection in C57BL/6J mice	Dreesen et al., 2014
Induction of IL-6	Early control of acute *G. lamblia* infection in C57BL/6J mice	Zhou et al., 2003
Induction of matrix metalloprotease 7 (Mmp7)	Production of mature α-defensins in C57BL/6J mice and control of *G. lamblia* infection	Tako et al., 2013
Induction of chemokines (CCL2, CCL20, CXCL1, CXCL2, and CXCL3)	Recruitment of host immune cells to the site of infection (?)	Roxström-Lindquist et al., 2005
Induction of stress-induced genes (c-Fos, c-Jun, and IER3)	Regulation of cell stress during *G. lamblia* infections in Caco-2 cells (?)	Roxström-Lindquist et al., 2005
Reduction of cell proliferation genes (G0S2, PCNA, ORC5L, MCM2, MCM3)	Response to NO production in Caco-2 cells infected with *G. lamblia*	Roxström-Lindquist et al., 2005
***Cell viability***		
Activation of caspase-3-dependent apoptosis	Assembled-specific induction of apoptosis by *G. lamblia*-infected HCT-8 cells and strain-dependent apoptosis of human duodenal epithelial cell line	Chin et al., 2002; Koh et al., 2013
Activation of caspase-3, caspase-8, and caspase-9-dependent apoptosis	Activation of both the intrinsic and the extrinsic apoptotic pathways of HCT-8 cells infected with *G. lamblia*	Panaro et al., 2007
Activation of sodium-dependent glucose cotransporter (SGLT)-1	Protection against *G. lamblia*-induced apoptosis in Caco-2 cell cultured in high glucose media	Yu et al., 2008
***TJs and cytoskeleton***		
Disruption of ZO-1	Increased permeability in HCT-8 and human duodenal epithelial cell line infected with *G. lamblia*	Buret et al., 2002; Chin et al., 2002; Koh et al., 2013
Reduced claudin-1 expression	Decreased absorption, increased ion secretion and villous shortening in duodenal biopsies from *G. lamblia* infected patients	Troeger et al., 2007
Relocation of claudin-1 and F-actin retraction	Increased paracellular permeability and microvilli atrophy in *G. lamblia*-infected Caco-2 cells	Maia-Brigagão et al., 2012
F-actin condensation and loss of perijunctional *α-actinin*	Increased permeability of Caco-2 and non-transformed human epithelial cell line (SCBN) infected with *G. lamblia*	Teoh et al., 2000
F-actin and ZO-1 reorganization	Myosin-light chain kinase (MLCK)-dependent increased cell permeability in *G. lamblia* infections	Scott et al., 2002

## Cryptosporidium parvum

*Cryptosporidium parvum* is an obligatory intracellular intestinal parasite of humans and animals and is responsible for many cases of cryptosporidiosis worldwide. Cryptosporidiosis is characterized as a watery diarrhea and is a potentially life-threatening disease in both immunocompetent and immunosuppressed hosts. The parasite exists in the environment as an oocyst that contains four sporozoites. When ingested by a host (by fecal-oral contact or by contaminated drinking or recreational water), the oocyst travels through the gut lumen to the small intestine where the sporozoites are released by excystation. Motile sporozoites then attach to the intestinal epithelium and are enveloped by the host cell apical membrane forming an extracytoplasmic parasitophorous vacuole inside which the parasite undergoes asexual multiplication. The sporozoites then enter a sexual reproductive stage and develop into female macrogamonts and male microgamonts. After fertilization, the zygote can develop into two types of oocysts: (a) a thick-walled oocyst that is excreted into the environment or (b) a thin-walled oocyst that can auto-infect the host (Petersen, 1993).

Cryptosporidiosis is characterized by watery diarrhea, malabsorption and wasting. The pathophysiology of cryptosporidiosis is multifactorial, and three pathophysiological mechanisms have been proposed to occur during infection: first, infiltration of the lamina propria by host immune cells (lymphocytes, macrophages, and neutrophils), responsible for inflammatory diarrhea; increased transepithelial permeability, villous atrophy, crypt hyperplasia and cell death, characteristic of exudative diarrhea; and malabsorption due to loss of the intestinal architecture relating to osmotic diarrhea. In the following section, the events preceding the development of diarrhea during *C. parvum* infection are described.

### Host Cell Transcription during *Cryptosporidium* Infection

As a member of the phylum Apicomplexa, which also includes *Plasmodium* and *Toxoplasma* sp., *C. parvum* is equipped with a specialized apical apparatus named the apical complex. This complex is composed of secretory organelles such as rhoptries, micronemes and dense granules, which play distinct roles during *Cryptosporidium*–host cell interaction. As an obligatory intracellular parasite, invasion of cells by *Cryptosporidium* is mandatory and is preceded by the adhesion of parasites to intestinal epithelial cells. Adherence allows the parasite to anchor itself to the epithelial layer, and this process has been shown to be mediated by the presence of adhesins like thrombospondins (TSPs) and thrombospondin-related adhesive protein (TRAP)-C1, and glycoproteins such as mucins and mucin-like proteins (GP900), to cite a few examples (Wanyri and Ward, 2006). Following adherence, parasites are internalized, resulting in subtle changes of host cell gene expression such as the development of a mechanism for evading the host cell immune response. Indeed, several studies have described changes in certain selected host cellular genes due to *Cryptosporidium* infection (Castellanos-Gonzalez et al., 2008; Zhou et al., 2009). For example, microarray analysis of human ileal mucosa explants infected with *C. parvum* or *C. hominis* demonstrated increased expression of osteoprotegerin (OPG) mRNA compared to uninfected cells (Castellanos-Gonzalez et al., 2008). The relevance of this finding was further extended, as jejunal biopsy specimens obtained from a volunteer (before and after experimental infection with *C. meleagridis*) displayed a 1281-fold increase in OPG mRNA post-infection (Castellanos-Gonzalez et al., 2008). OPG is a soluble glycoprotein produced by osteoblasts, intestine cells, hematopoietic and immune cells (dendritic cells and lymphocytes; Simonet et al., 1997; Vidal et al., 2004). It is a member of the TNF superfamily which includes proteins such as TNF-α, Fas/FasL and TRAIL, which are involved in cell differentiation, proliferation, survival, apoptosis and in immune responses (Simonet et al., 1997). Various human intestinal epithelial cell lines were reported to constitutively express OPG, especially during inflammation, suggesting that it may play an important role as a mucosal immunoregulatory factor (Vidal et al., 2004). Whether OPG exerts its biological effect in response to cell inflammation during *C. parvum* infection remains to be elucidated. Nonetheless, data obtained by Castellanos-Gonzalez et al. (2008) demonstrated that OPG is produced during the early stages of *C. parvum* infection blocking TRAIL-mediated apoptosis of host cells, indicating OPG as a protective factor in cryptosporidiosis.

Cytokines and chemokines are proteins that regulate inflammation and modulate cellular activities such as growth, survival and differentiation (Zlotnik, 2000; Dinarello, 2007). They may act as pro- or anti-inflammatory factors (cytokines) or as chemotactic attractants (chemokines) to leukocytes and in trafficking of immune cells (Dinarello, 2007). They can be produced by a series of cells such as T helper cells (Th) and macrophages but also by intestinal epithelial cells (Stadnyk, 2002). Reports on the pathogenesis of cryptosporidiosis have shown increased mRNA levels for cytokines (IL-1β, IL-4, IL-8, IL-14, IL-15, IFN-γ, TGF-β) and chemokines (C-C and C-X-C subfamilies) in human and murine intestinal cells and xenografts infected with *C. parvum* (Laurent et al., 1997; Seydel et al., 1998b; Robinson et al., 2000, 2001; Lacroix-Lamandé et al., 2002; Deng et al., 2004; Tessema et al., 2009). These results are consistent with previous observations showing the recruitment of effector cells to the site of inflammation in the intestinal lamina propria from *Cryptosporidium*-infected patients (Robinson et al., 2001), as chemokines may control the localization of immune cells throughout the body (Griffith et al., 2014). Therefore, these data suggest that expression of multiple cytokines and chemokines by host cells may play an important role in the control of inflammation during *Cryptosporidium* infections. More recently, fractalkine or CX3CL1, a membrane-bound chemokine of the CX3C family, was shown to be upregulated in human biliary epithelial cells following *C. parvum* infection. Induction of CX3CL1 expression involved downregulation of microRNAs (miR-424 and miR-503) both known to target the CX3CL1 3′ UTR, suppressing its translation and inducing RNA degradation (Zhou et al., 2013). MicroRNAs are non-coding, single-stranded RNAs that negatively regulate gene expression through interactions with 3′ UTRs of the target mRNA (Bartel, 2009).

Defensins are a family of antimicrobial peptides expressed by different cells including Paneth cells in the epithelium of the small intestine. They are subdivided into two families, the α-defensins (also known as cryptdins in mice) and the β-defensins, both displaying microbicidal activity (Bevins and Salzman, 2011). Experiments with human colonic (HT29) and murine rectal adenocarcinoma (CMT-93) cell lines infected with *C. parvum* have shown differential β-defensin gene expression (Zaalouk et al., 2004). Indeed, using reverse transcription-PCR, a reduction in human-defensin-1 (hBD-1) and induction of hBD-2 were observed in *Cryptosporidium*-infected colonic cells. Furthermore, enterocytes infected with *C. parvum* and treated with recombinant hBD-1 and hBD-2 showed a reduction in the percentage of viable sporozoites, indicating that these peptides may have an important role in the host’s innate response against infection (Zaalouk et al., 2004). Upregulation of inducible nitric oxide synthase (iNOS) was also shown to occur in neonatal piglets during acute *C. parvum* infection. Curiously, expression of iNOS was not restricted to infected cells, possibly indicating a non-specific response against *Cryptosporidium* infection, although the importance of iNOS in the control of tissue parasitism was further confirmed, as inhibition of iNOS activity resulted in increased parasite burden in intestinal epithelial cells (Gookin et al., 2006). Finally, iNOS induction was shown to be NF-κB dependent, as iNOS activity was abolished when infected cells were incubated with lactacystin (a proteasome inhibitor that prevents degradation of IκBα; Gookin et al., 2006). These data, together with the demonstration that iNOS expression by macrophages and other cell types occurs in tissues from patients with a wide variety of infectious diseases (Bogdan, 2001), may suggest a protective role for nitric oxide in cryptosporidiosis. Further experiments are needed to confirm this hypothesis in the human disease.

### *Cryptosporidium parvum* Infection and Host Cell Death

The first report on the occurrence of apoptosis in *C. parvum*-infected human biliary epithelial cells (H69 cells) was issued by Chen et al. (1998). Later, nuclear condensation and DNA fragmentation (as markers of apoptosis) during *C. parvum* infection were shown to be caspase-dependent and induced by Fas/FasL, as caspase inhibitors or neutralizing antibodies to either the Fas receptor (Fas) or Fas ligand (FasL) blocked these events (Chen et al., 1999; Ojcius et al., 1999). *Cryptosporidium*-induced apoptosis was documented to occur independent of cell line (CaCo-2, MDBK, and HCT-8 cells) and resulted in impaired *C. parvum* development *in vitro*, suggesting a host–cell mechanism to control the spread of infection (Widmer et al., 2000). Further studies, however, demonstrated that apoptotic changes of intestinal epithelial cells were modulated by the *C. parvum* development stages and displayed biphasic activation with early inhibition (at the trophozoite stage) and late moderate promotion (at the sporozoite and merozoite stages; Mele et al., 2004; Liu et al., 2009). On the basis of these data, it has been suggested that *Cryptosporidium* precisely regulates host cell apoptosis to favor its growth and development at initial stages of infection, and to promote its propagation later on.

In line with this hypothesis, namely that *Cryptosporidium* can exert control on the processes that regulate apoptosis in the host, Chen et al. (2001) have shown that *C. parvum*-infected biliary cells activated the NF-κB signaling cascade, leading to secretion of the pro-inflammatory cytokine IL-8 and inhibition of cell apoptosis. Moreover, these events were restricted to infected cells given that *C. parvum*-induced apoptosis was limited to bystander uninfected cells (Chen et al., 2001). Inhibition of host cell apoptosis during *Cryptosporidium* infection has also been reported to involve the expression of members of the IAP family (inhibitors of apoptosis proteins) such as c-IAP1, c-IAP2, XIAP, and survivin. In this case, it was demonstrated that knockdown of survivin (but not that of c-IAP1, c-IAP2, or XIAP) by siRNA enhanced caspase-3/7 activity and resulted in increased host cell apoptosis and decreased *C. parvum* infection (Liu et al., 2008). The role of IAPs in the control of cell death in cryptosporidiosis is reinforced by a study showing that XIAP mediated proteasome-dependent inhibition of activated caspase-3 in *C. parvum* infection (Foster et al., 2012).

### Disassembly of Tight Junctions during *Cryptosporidium* Infection

Dysfunction of the epithelial barrier during *in vivo* intestinal infections with *Cryptosporidium* has been documented in humans and animals. Villous atrophy, hyperplasia of the crypt epithelium and increased transepithelial permeability are some of the abnormalities reported in cryptosporidiosis (Genta et al., 1993; Adams et al., 1994; Gookin et al., 2002). *In vitro* studies on *Cryptosporidium andersoni*-infected human (Caco-2) and bovine (MDBK and NBL-1) epithelial cells reported disruption of ZO-1 and nuclear fragmentation during infection (Buret et al., 2003). Interestingly, both events were reversed by pretreatment of host cells with recombinant human epidermal growth factor (rhEGF), and significantly reduced infection rates in bovine and human enterocytes (Buret et al., 2003). The relationship, if any, between *C. andersoni*-induced ZO-1 disruption and loss of barrier function is still unknown. Moreover, how EGF exerts its biological effect during *Cryptosporidium* infection deserves further investigation. **Table 2** summarizes work on *Cryptosporidium*.

**Table 2:** Summary on studies describing the effect of *Cryptosporidium* infection on host cell responses.

Target on host cells	Effect	Reference
***Gene transcription***		
Induction of osteoprotegerin (OPG)	Immune modulation of host cell response to *C. parvum* and *C. hominis* infection of human ileal explants	Castellanos-Gonzalez et al., 2008
Induction of cytokines (IL-1β, IL-4, IL-8, IL-14, IL-15, IFN-γ, TGF-β)	Control of inflammation in human intestinal xenografts, jejunal biopsies, C57BL/6 mice and HCT-8 cells infected with *C. parvum*	Laurent et al., 1997; Seydel et al., 1998b; Robinson et al., 2000, 2001; Lacroix-Lamandé et al., 2002; Deng et al., 2004; Tessema et al., 2009
Induction of C-X-C chemokines	Recruitment of immune cells to the *lamina propria* of *C. parvum*-infected cells	Laurent et al., 1997
Induction of fractalkine or CX3CL1 chemokine	Donwregulation of microRNAs and activation of mucosal antimicrobial defense against *C. parvum* in human biliary epithelial cells	Zhou et al., 2013
Modulation of β-defensin expression	Control of host innate immune response in *C. parvum*-infected HT29 cells	Zaalouk et al., 2004
Induction of nitric oxide synthase (iNOS)	Control of tissue parasitism in neonatal piglets and human epithelial cells infected with *C. parvum*	Gookin et al., 2006
***Cell viability***		
Activation of caspase-3-dependent signaling cascade	Induced Fas/FasL-dependent apoptosis in *C. parvum*-infected biliary epithelial cells	Chen et al., 1999; Ojcius et al., 1999
Expression of survivin	Protection against *C-parvum*-induced caspase 3/7 apoptosis in HCT-8 cells	Liu et al., 2008
Activation of XIAP	Proteasome-dependent inhibition of activated caspase-3 and cell apoptosis in piglets infected with *C. parvum*	Foster et al., 2012
***TJs and cytoskeleton***		
Disruption of ZO-1	Unknown effect in *C. andersoni*-infected Caco-2 and MDBK cells	Buret et al., 2003

## Entamoeba histolytica

*Entamoeba histolytica* is a protozoan parasite that colonizes the large intestine of humans causing amoebiasis. Although most infections with *E. histolytica* are asymptomatic, some patients may experience clinical manifestations of invasive amoebiasis such as amoebic colitis and amoebic liver abscess (Marie and Petri, 2014).

*Entamoeba histolytica* has a simple lifecycle that involves two distinct morphogenetic stages, the amoeboid and proliferative trophozoite, and the infectious cyst form. Human infections begin with ingestion of the viable cysts in food or water that has been contaminated by feces. Excystation occurs in the small intestine, and released trophozoites migrate to the colon where they multiply by binary fission. In the end, trophozoites encyst, completing the lifecycle when they are excreted into the environment in stool (Marie and Petri, 2014). Trophozoite adhesion to colonic mucus and epithelial cells is a critical step in the colonization of the large intestine by *E. histolytica* and a Gal/GalNAc lectin (260 kDa) expressed at the parasite surface was shown to mediate its binding to host mucins and cell surface carbohydrates (Frederick and Petri, 2005).

### Host Cell Transcription during *Entamoeba histolytica* Infection

It is well-known that infection with the protozoan parasite *E. histolytica* results in significant inflammatory responses that contribute to tissue damage and invasion. *In vitro* studies using co-culture of human epithelial and stromal cells and cell lines (HeLa, HT29, and T84) demonstrated an upregulation of IL-8 transcripts during *E. histolytica* infections that correlated with increased secretion of this pro-inflammatory cytokine and others such as GROα, GM-CSF, IL-6, and IL-1α (Eckmann et al., 1995). Increased mRNA for both IL-1β and IL-8 were also reported to occur *in vivo* when mouse–human intestinal xenografts (SCID-HU-INT) were infected with *E. histolytica* trophozoites (Seydel et al., 1997). The relevance of these findings was further reinforced when intraluminal administration of an antisense oligonucleotide (to block the production of IL-1β and IL-8) inhibited the gut inflammatory response to *E. histolytica* infection (Seydel et al., 1998a).

In human patients with acute or convalescent amoebiasis, gene expression profiles obtained by microarray analysis of intestinal biopsies clearly demonstrated upregulation of *REG1A* and *REG1B* genes (Peterson et al., 2011). *REG1A* and *REG1B* belong to the regenerating islet-derived (REG) gene family encoding for C-type lectin-like proteins (Parikh et al., 2012). They are involved in the proliferation and differentiation of diverse cell types and are well-known to be highly expressed in some pathologies as inflammatory diseases, cancer and diabetes (Parikh et al., 2012). One member of this family, the REG1α protein, was also shown to mediate the anti-apoptotic effect of STAT3 in cancer cells (Sekikawa et al., 2008). During *E. histolytica* infections, expression of REG1α and REG1β were shown to inhibit parasite-induced apoptosis *in vitr*o as REG1-/- mice were found to be more susceptible to cell death.

### *Entamoeba histolytica* Infection and Host Cell Death

As the name suggests, *E. histolytica* (*histo*: tissue and *lytica*: destroyer) is a tissue-destroying amoeba (Pinilla et al., 2008) and host cell apoptosis is one of the most common events associated with infections with this parasite (Huston et al., 2000; Christy and Petri, 2011; Marie and Petri, 2014). The mechanisms leading to host cell death in *E. histolytica* infections are not completely understood. However, apoptosis and trogocytosis have been reported to occur in amoebiasis without parasite penetration within host cells. Apoptosis was first suggested to occur during *E. histolytica* infections as DNA fragmentation was observed after trophozoite adhesion to a murine myeloid cell line (FDC-P1; Ragland et al., 1994). Cell killing by *E. histolytica* was further shown to occur via a Bcl-2-independent mechanism, as FDC-P1 cells transfected with a retrovirus construct to express the Bcl-2 protein were susceptible to amoeba contact-dependent killing (Ragland et al., 1994). Later, using Jurkat cells it was demonstrated that infection with *E. histolytica* rapidly activated caspase-3, independently of caspase-8 and -9 activation (Huston et al., 2000). Interestingly, *E. histolytica* activation of caspase-3 was followed by phagocytosis of host cells, suggesting that cell killing precedes ingestion by trophozoites (Huston et al., 2003). Over the past years, diverse studies have tried to elucidate the pathways explored by *E. histolytica* to trigger host cell apoptosis. For example, in hepatocytes, live *E. histolytica* was shown to induce an apoptosis-like death without the participation of both Fas and TNF-α pathways (Seydel and Stanley, 1998). Teixeira and Mann have observed that adhesion of *E. histolytica* trophozoites to Jurkat cells induced a contact-dependent protein dephosphorylation by host cell protein tyrosine phosphatases (PTPs) such as SHP-1 and SHP-2 (Kim et al., 2010), since pretreatment of cells with a PTP inhibitor inhibited amoeba-induced dephosphorylation and cell apoptosis (Teixeira and Mann, 2002). Activation of host cell PTP occurred through a calcium-dependent calpain protease responsible for PTP1B cleavage that led, at last, to cell death (Teixeira and Mann, 2002). Reinforcing these data, activation of host cell calpain by *E. histolytica* was also observed by others (Kim et al., 2007; Jang et al., 2011) and was shown to modulate the degradation of STAT proteins (STAT3 and STAT5) and NF-κB (p65) in Caco-2 cells (Kim et al., 2014). Furthermore, pretreatment of Caco-2 cells with calpeptin (a calpain inhibitor) or calpain silencing partially reduced *Entamoeba*-induced DNA fragmentation (Kim et al., 2014).

In recent years, a number of studies have shown that oxidative stress could cause cellular apoptosis via both the extrinsic and intrinsic pathways in health and pathological conditions (Lin and Beal, 2006). In amoebiasis, for example, incubation of human neutrophils with *E. histolytica* trophozoites triggered NADPH oxidase-dependent production of reactive oxygen species (ROS) and cell apoptosis (Sim et al., 2005). The mechanism involved in *Entamoeba*-induced ROS generation and apoptosis was associated with ERK1/2 activation, possibly through β2-integrin, as cells pretreated with a MEK1 inhibitor (PD98059) and with a monoclonal antibody to CD18 (anti-integrin β2 subunit) prevented cell apoptosis (Sim et al., 2005, 2007). Phosphatidylinositol-3-kinase (PI-3-kinase) was also involved in ROS production and apoptosis during *Entamoeba* infection, suggesting that signaling molecules may be key factors in *E. histolytica*-induced, ROS-dependent apoptosis (Sim et al., 2007). Similar results were reported in colonic Caco-2 and HT-29 cells, as increased levels of intracellular ROS were reported to occur through NOX1 oxidase after cell exposure to trophozoites (Kim et al., 2011, 2013). In this case, cell death was shown to be caspase-independent and the signaling cascade activated during this event is still unknown.

The historical concept that *E. histolytica* kills cells by apoptosis was recently challenged by Ralston et al. (2014). Using both Caco-2 and Jurkat cells, the authors demonstrated that, immediately after contact with human cells, *E. histolytica* ingests small fragments of the cell membrane, some containing cellular components like cell cytoplasm and mitochondria (Ralston et al., 2014). Surprisingly, host cells were alive when ingestion of fragments was initiated, and resulted in the elevation of the intracellular amount of calcium before the eventual death of cells as trophozoites detached from corpses. The internalization of cell fragments by *E. histolytica* was named as amoebic trogocytosis (from the Greek *trogo*, for nibble) and only occurred with live cells as pre-killed cells are ingested intact (Ralston et al., 2014).

On the whole, the results of these studies demonstrated that *E. histolytica* infections might result in cell death both by apoptosis and trogocytosis, and that these events might contribute to tissue invasion by the parasite.

### Disassembly of Tight Junctions during *Entamoeba* Infection

It is widely accepted that tissue invasion by *E. histolytica* is preceded by the interaction of trophozoites with intestinal epithelial cells, and a series of studies have shown that this interaction impacts cell morphology, intercellular contacts and regulation of paracellular transport of molecules across the intestinal epithelium. Indeed, *in vitro* studies have shown a rapid decrease in transepithelial resistance (TER) and increased mannitol flux during trophozoite interaction with polarized human intestinal Caco-2 and T84 cells (Martinez-Palomo et al., 1985; Li et al., 1994; Leroy et al., 2000). Apical injury of host cells such as loss of brush border in regions of contact between epithelial cells and amoebae was also reported. Importantly, these changes were only observed when Caco-2 cells were co-incubated with live trophozoites but not with amoeba lysates or conditioned medium, indicating that they were not mediated by soluble amoebic cytotoxins (Li et al., 1994; Leroy et al., 2000). In human enteric T84 cells co-cultured with amoebae, decreased TER was associated with changes in TJ proteins like release of ZO-1 from ZO-2, degradation of ZO-1 and dephosphorylation of ZO-2 (Leroy et al., 2000).

Besides *E. histolytica*-host cell contact, amoebic products also have been shown to be crucial for cellular barrier dysfunctions during parasite infections. For example, prostaglandin E_2_ (PGE_2_) secreted by *E. histolytica* was shown to alter the spatial localization of claudin-4 that resulted in increased sodium ion permeability through TJs (Lejeune et al., 2011). EhCP112, an *E. histolytica*-secreted cysteine protease, has been shown to digest gelatin, collagen type I, fibronectin, hemoglobin and, most importantly, to destroy MDCK cell monolayers (Ocádiz et al., 2005). When complexed with EhADH112 adhesin, the formed EhCPADH112 complex was shown to co-localize with claudin-1 and occludin at the TJs after the incubation of epithelial MDCK cells with trophozoite extracts. Furthermore, EhCPADH112 induced progressive disruption of the paracellular barrier as measured by TER. Importantly, these effects were reversed when co-cultures were incubated with a protease inhibitor cocktail or a monoclonal antibody against the EhCPADH112 complex (Betanzos et al., 2013). Cysteine proteases are important virulence factors in *E. histolytica*, and 20 genes encoding for these proteases have been identified on the genome (Bruchhaus et al., 2003). Their role in parasite loss of host cell integrity is highlighted in a study showing that calpain (a calcium-dependent cysteine protease) activation resulted in degradation of paxillin, Cas, vimentin, vinculin, talin, and α- or β-spectrin in Jurkat T cells infected with *E. histolytica* (Lee et al., 2011). In addition to proteases, Goplen et al. (2013) have shown that *E. histolytica* expressed a cognate “occludin-like” protein of the host, as revealed by confocal microscopy using antibodies for human occludin. Apical administration of “occluding-like” protein to T84 human colonic epithelial cells resulted in epithelial disruption and decreased TER, suggesting the involvement of this protein in the pathophysiology of amoebiasis. The exact mechanism by which “occluding-like” protein exerts its effects is not completely understood but the authors suggested that it might compete for epithelial occludin–occludin interactions, a hypothesis that needs further investigation. Studies on *E. histolytica* are summarized in **Table 3**.

**Table 3:** Summary on studies describing the effect of *Entamoeba histolytica* infection on host cell responses.

Target on host cells	Effect	Reference
***Gene transcription***		
Induction of cytokines (GROα, GM-CSF, IL-6, IL-8, IL-1α, and IL-1β)	Control of inflammation in *E. histolytica*-infected human cells (HeLa, HT29, and T84) and mouse-human intestinal xenografts	Eckmann et al., 1995; Seydel et al., 1997, 1998a
Induction of REG1A and REG1B	Inhibition of parasite-induced apoptosis in colonic biopsies of *E. histolytica*-infected patients	Peterson et al., 2011
***Cell viability***		
Activation of caspase-3 signaling pathway	Induction of caspase-8 and caspase-9 independent apoptosis of Jurkat cells	Huston et al., 2003
Induction of “apoptosis-like” mechanisms	Death of hepatocytes in Fas and TNF-α independent pathways	Seydel and Stanley, 1998
Dephosphorylation of host cell proteins by PTPs	Induction of calcium-dependent calpain protease and apoptosis of *E. histolytica*-infected Jurkat cells	Teixeira and Mann, 2002
Activation of calpain	Cell death of HT-29 and Jurkat cells infected with *E. histolytica* and modulation of STAT proteins and NF-κB DNA fragmentation	Kim et al., 2007, 2014; Jang et al., 2011
Activation of NADPH-oxidase	Induction of ERK1/2 pathways and ROS-dependent apoptosis of human neutrophils infected with *E. histolytica*	Sim et al., 2005, 2007
Activation of NOX1 oxidase	Production of ROS and caspase-independent apoptosis of Caco-2 and HT-29 cells infected with *E. histolytica*	Kim et al., 2011, 2013
Activation of PI-3-K	ROS-mediated neutrophil apoptosis induced by *E. histolytica*	Sim et al., 2007
Ingestion of host cell membrane fragments by trophozoites	Elevation of intracellular Ca^2+^ and death of cells by trogocytosis	Ralston et al., 2014
***TJs and cytoskeleton***		
Trophozoite interaction with polarized cells	Reduction in transepithelial resistance and increased mannitol flux in *E. histolytica*-infected Caco-2 and T84 cells	Li et al., 1994; Leroy et al., 2000
Degradation of ZO-1, release of ZO-1 from ZO-2, and dephosphorylation of ZO-2	Reduction in transepithelial resistance and increased mannitol flux in *E. histolytica*-infected T84 cells	Leroy et al., 2000
Relocalization of claudin-4	Increased sodium ion permeability in amoeba infected T84 cells	Lejeune et al., 2011
Secretion of an “occludin-like” molecule by trophozoites	Disruption of epithelial barrier and reduction in transepithelial resistance in *E. histolytica*-infected T84 cells	Goplen et al., 2013

## Giardiasis, Cryptosporidiosis, And Amoebiasis: Mechanistic Similarities And Differences

The pathophysiology of diarrhea caused by *G. lamblia*, *Cryptosporidium* sp., and *E. histolytica* is multifactorial and, despite depending on the microbiological agent causing it, these diseases share some mechanistic features (**Table 4**).

**Table 4:** Pathophysiological mechanisms implicated in diarrhea caused by *G. lamblia*, *Cryptosporidium* sp., and *E. histolytica.*

Pathophysiological mechanism	Giardiasis	Cryptosporidiosis	Amoebiasis
Osmotic diarrhea	Malabsorption of nutrients was described to occur in response to reduced disaccharidase activity in the gut (Troeger et al., 2007)	Impaired absorptive function was shown to result in reduced absorption of both monosaccharides and co-transport of glucose-Na^+^ (Argenzio et al., 1990; Farthing, 2000)	Lactose malabsorption was reported in amoeba-infected patients (Rana et al., 2004)
Secretory diarrhea	Loss of epithelial absorptive surface (villous and microvilli atrophy) and chloride secretion were reported in colonic cells *in vitro*, in animal models and human patients (Gorowara et al., 1992; Scott et al., 2000; Troeger et al., 2007)	Damage to the absorptive villous and unbalanced secretory crypts were involved in electrolyte secretion. An unknown cryptosporidial enterotoxin was suggested to trigger net secretion (Guarino et al., 1995, 1997)	Increased mannitol flux and movement of Na^+^ ions into the intestinal lumen (Leroy et al., 2000; Lejeune et al., 2011)
Exudative diarrhea	Disruption or relocation of tight junctions proteins and dysfunctional epithelial barrier were associated with leak flux diarrhea (Troeger et al., 2007; Maia-Brigagão et al., 2012)	Disruption of epithelial tight junction, loss of intestinal barrier, dysregulated influx of immune and inflammatory cells and cell death by apoptosis were related to increased flux into the lumen (White, 2010)	Dysregulation of the TJ protein complex, decreased transepithelial resistance and cell apoptosis were associated with water flow (Ragland et al., 1994; Leroy et al., 2000; Betanzos et al., 2013)
Inflammatory diarrhea	Inflammation was rarely observed in chronically infected patients (Hanevik et al., 2007; Kohli et al., 2008)	Parasite products and infiltration of host immune cells in the *lamina propria* were associated to pathogenesis (Laurent et al., 1999)	Production of inflammatory mediators were correlated to tissue damage in amoebic diarrhea (Seydel et al., 1997, 1998a,b)
Motility problems	Malabsorption of nutrients, water-impaired absorption and electrolyte secretion were suggested to contribute to increased intestinal transit and peristalsis (Cotton et al., 2011)	Intestinal epithelial cell layer breakdown was shown to result in increased intestinal transit (Sharpstone et al., 1999; Brantley et al., 2003)	Not reported

The exact mechanism leading to giardiasis is unknown, although research points to a combination between osmosis, active secretion, exudation, inflammation and altered motility as drivers of *Giardia*-induced diarrhea. In molecular terms, disruption, reduced expression and/or relocation of TJ and cytoskeleton proteins (such as ZO-1, claudin-1, F-actin, and α-actinin) were shown to result in increased intestinal permeability and a drop in TER, indicating that infection can cause paracellular leakage (exudative diarrhea). The events leading to this class of diarrhea are similar to cryptosporidiosis and amoebiasis. Accordingly, disruption of ZO-1 was reported in colonic cells infected with *Cryptosporidium* (Buret et al., 2003) while in cells infected with *E. histolytica*, contact-dependent degradation of TJ proteins ZO-1 and ZO-2, dephosphorylation of ZO-2, relocation of claudin-4 and reduction in TER were shown to underlie exudative diarrhea (Leroy et al., 2000; Lejeune et al., 2011). However, while *Giardia* causes TJ disruption without penetrating the epithelium, *E. histolytica* kills (through apoptosis and trogocytosis), invades and destroys host tissues. In cryptosporidiosis, further studies are needed to assess whether cell invasion or parasitic products initiate these alterations.

Tight junction alterations were also observed to indirectly increase the luminal Cl^-^ concentration (secretory diarrhea) as a consequence of the loss of absorptive function (villous shortening, microvilli atrophy and increased cell death) and/or increased secretion (destruction of the epithelial barrier) in *Giardia*-infected cells (Troeger et al., 2007; Maia-Brigagão et al., 2012). Similarly, damage to the absorptive villi and enhanced fluid secretion from the crypts have been documented in cryptosporidiosis, supporting diarrhea by active secretion (Guarino et al., 1995, 1997). In amoebiasis, increased mannitol flux and movement of sodium ions into the intestinal lumen were reported (Leroy et al., 2000; Lejeune et al., 2011).

As digestion of nutrients in the small intestine depends on hydrolytic enzymes (disaccharidases such as sucrose, maltase, lactase, and peptidase) produced by the brush border membrane of microvilli, dysfunctional microvilli may interfere significantly with the absorption of nutrients. In giardiasis, loss of microvilli brush border, combined with villous atrophy, is responsible for disaccharidase insufficiencies and malabsorption of nutrients, ultimately causing osmotic diarrhea (Buret, 2007, 2008; Troeger et al., 2007). Likewise, in enteric cryptosporidiosis, villous atrophy and crypt hyperplasia were shown to account for impaired monosaccharide and glucose-Na^+^ absorption while lactose malabsorption was described in individuals infected with *E. histolytica* (Rana et al., 2004).

In some diarrheal infections, the association between impaired absorption and increased secretion may contribute to accelerated intestinal transit. Indeed, in giardiasis and cryptosporidiosis, increased motility was reported, which in turn may contribute to the exacerbation of weight loss observed in *Giardia*-infected patients. On the contrary, whether motility dysfunction occurs and its importance on the development of amoebiasis it are unclear. However, it cannot be ruled out, as increased secretion and malabsorption are triggered by *E. histolytica* infection.

Despite the similarities in the events leading to osmotic, secretory and exudative diarrhea, there are some differences between giardiasis, cryptosporidiosis, and amoebiasis when considering the immunological and inflammatory response of the host (inflammatory diarrhea). For example, while several lines of evidence support the hypothesis that infections with *Giardia* are rarely accompanied by inflammation (Hanevik et al., 2007; Morken et al., 2008), a parasite extract was shown to be a poor cytokine inducer (inducing only small amounts of IL-6 and TNF-α; Zhou et al., 2003, 2007). On the contrary, a hallmark of amoebiasis and cryptosporidiosis is acute intestinal inflammation dominated by NF-κB-mediated secretion of inflammatory cytokines produced by host cells (Eckmann et al., 1995; Seydel et al., 1997; McCole et al., 2000; Chen et al., 2001; Hou et al., 2010). For example, IL-1β, IL-6, IL-8, TNF-α, and IFN-γ are key factors in the inflammatory response elicited by host cells after contact with amoebae (Eckmann et al., 1995; Seydel et al., 1997; Hou et al., 2010). However, whether production of pro-inflammatory cytokines influences the permeability of epithelial cell TJs and gut absorption is not known. Similar to amoebiasis, upon *Cryptosporidium* infection, epithelial cells release pro-inflammatory cytokines (IL-1β, IL-8, TNF-α, IFN-γ) and chemokines (C-X-C and fractalkine) to the site of infection, which in turn may contribute to increased epithelial permeability, impaired intestinal absorption and enhanced secretion (Seydel et al., 1998a; Farthing, 2000; Lacroix-Lamandé et al., 2002).

Collectively, these observations suggest that malabsorption, secretion of electrolytes and impairment of TJs may underlie luminal fluid accumulation during *G. lamblia* infection. Marked mucosal inflammation, decreased absorptive surface and malabsorption are thought to contribute to the pathogenesis of *Cryptosporidium*-induced diarrhea, while in *E. histolytica*-infected cells, epithelial destruction and inflammation infection appears to be the basis of the disease.

## Concluding Remarks

Intestinal parasitism is extremely common, with *G. lamblia*, *C. parvum*, and *E. histolytica* being the most important intestinal protozoan parasites of humans worldwide. Infections begin when a person ingests the infective stage of the parasite with contaminated food or water. Once inside the host, parasites lodge in the intestinal tract causing acute and self-limited diarrhea. However, in some patients, the disease can progress to chronic diarrhea and related complications such as malnutrition, growth delays and cognitive impairment.

Significant progress has been made in understanding the processes by which *G. lamblia*, *C. parvum*, and *E. histolytica* trigger diarrhea and how the host cell responds to infection. Disruption of TJ barrier function, alterations of host cell architecture, and transcription of genes involved in host immunity and cell death are some of the events elicited in the host cell when interacting with these parasites.

Future elucidation of the processes that integrate these events and eliminate the disease may lead to novel therapeutic approaches for diarrhea caused by enteropathogenic parasites.

## Author Contributions

Conceived and wrote the paper RT. Wrote the paper BG.

## Conflict of Interest Statement

The authors declare that the research was conducted in the absence of any commercial or financial relationships that could be construed as a potential conflict of interest.
